# Chemical Influence of Carbon Interface Layers in Metal/Oxide Resistive Switches

**DOI:** 10.1021/acsami.3c00920

**Published:** 2023-03-29

**Authors:** Deok-Yong Cho, Ki-jeong Kim, Kug-Seung Lee, Michael Lübben, Shaochuan Chen, Ilia Valov

**Affiliations:** † IPIT and Department of Physics, Jeonbuk National University, Jeonju 54896, Republic of Korea; ∥ Pohang Accelerator Laboratory, Pohang 37673, Republic of Korea; # Peter Gruenberg Institute, Research Centre Juelich, Juelich 52425, Germany; ‡ IWE2, RWTH Aachen University, Sommerfed strasse 24, Aachen 52074, Germany; § Institute of Electrochemistry and Energy Systems “acad. E. Budewski”, Bulgarian Academy of Sciences, “acad. G Bonchev” street Bl.10, Sofia 1113, Bulgaria

**Keywords:** carbon, Ta_2_O_5_/Ta, resistive switch, X-ray absorption spectroscopy, X-ray photoelectron spectroscopy

## Abstract

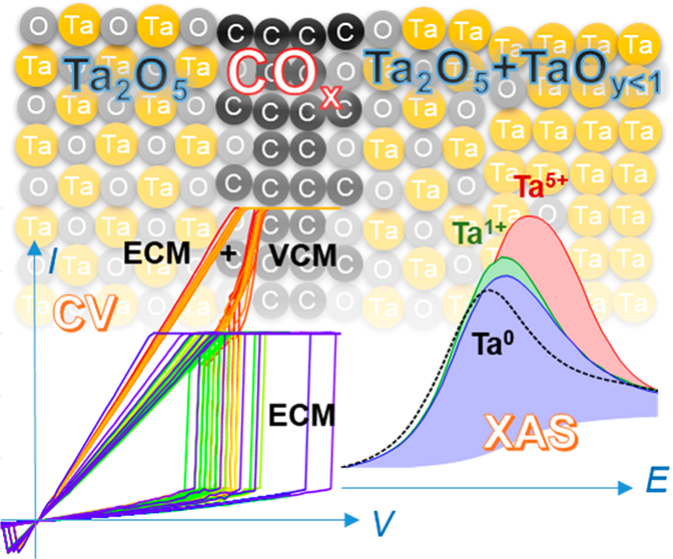

Thin layers introduced between a metal electrode and a solid electrolyte can significantly alter the transport of mass and charge at the interfaces and influence the rate of electrode reactions. C films embedded in functional materials can change the chemical properties of the host, thereby altering the functionality of the whole device. Using X-ray spectroscopies, here we demonstrate that the chemical and electronic structures in a representative redox-based resistive switching (RS) system, Ta_2_O_5_/Ta, can be tuned by inserting a graphene or ultrathin amorphous C layer. The results of the orbitalwise analyses of synchrotron Ta L_3_-edge, C K-edge, and O K-edge X-ray absorption spectroscopy showed that the C layers between Ta_2_O_5_ and Ta are significantly oxidized to form CO_*x*_ and, at the same time, oxidize the Ta layers with different degrees of oxidation depending on the distance: full oxidation at the nearest 5 nm Ta and partial oxidation in the next 15 nm Ta. The depth-resolved information on the electronic structure for each layer further revealed a significant modification of the band alignments due to C insertion. Full oxidation of the Ta metal near the C interlayer suggests that the oxygen-vacancy-related valence change memory mechanism for the RS can be suppressed, thereby changing the RS functionalities fundamentally. The knowledge on the origin of C-enhanced surfaces can be applied to other metal/oxide interfaces and used for the advanced design of memristive devices.

## Introduction

Resistive switching (RS) is a phenomenon in which the electrical conductance of a substance changes reversibly depending on external bias. From a microscopic point of view, the resistance change is caused by the collective motions of charge carriers (ions and/or electrons), changing the resistance of a certain volume or of an electrode/electrolyte interface.^1−7^ This process can involve in many cases constituting or breaking conducting filaments. Thus, the electrochemical interaction between the charge carrier(s) and the matrix is of highest importance in determining the functionality and characteristics of the entire RS device.^8−14^

It has been demonstrated that 2–3-nm-thick native layers are formed at the metal/oxide interfaces, and these layers can significantly change the kinetics of the electrochemical processes and modify the memristive performance and functionalities.^15,16^ In many cases, the influence of such interfacial layers is decisive because they can either benefit the RS process by providing, e.g., ions, or can be inhibiting by forming ionic and/or electronic barriers. To enhance the advantageous effects of intermediate layers and suppress the formation of native films (as a source of stochasticity), 2D materials or 2–3 nm thin films have been inserted at the electrode/electrolyte interfaces.^17−28^ In the cases of both electrochemical metallization memory (ECM or conductive bridge random access memory, CBRAM) and valence change memory (VCM or oxide random access memory, OxRAM), this approach leads to a significant change and improvement of the properties and performance of the devices. A special case is represented by inserting C layers at the insulator/metal interface, which changes the characteristics of RS.^18,29^ The efficacy of the C layers has been studied extensively due to the discovery of various nanoscale architectures including graphite,^30,31^ carbon nanotubes,^32,33^ and graphene.^29,34,35^ According to our previous study, the presence of graphene or amorphous C at the interface of a bipolar resistive switch, comprised of an insulating Ta_2_O_5_ and a Ta metal, seems to serve as a physicochemical barrier to inhibit the electrochemical reactions and ionic movements of O^2−^ across the interface.^29^ Nevertheless, the exact mechanism of how the C layers are acting and interacting with metal electrode and oxide film is still unclear. For example, whether they are chemically inert and represent merely physical barriers or are active so as to bond and/or mediate ions. Understanding the nature of the C interlayer effects and the mechanism of action will allow better design and control of the electrochemical processes and ionic movements during RS and in general improve the device performance. To clarify the microscopic origin of this effect, it is essential to analyze the chemical and electronic structure of the C-added interfaces in the oxide/metal RS systems.

One significant obstacle to characterize the oxide/metal interface in the RS devices using conventional electron-beam techniques such as transmission electron microscopy or Auger electron spectroscopy is that the chemistry of the RS material systems is vulnerable particularly to charge injections by the electron beams for the measurement itself. The injected charges can locally alter the RS states because the electric field is the very source of ionic movements for constituting or breaking the conducting filaments. Thus, a spectroscopic methodology using a charge-neutral source, e.g., light or a neutron, is desirable to examine the chemical states as is with minimal perturbation.^36,37^ In particular, the use of X-rays is suitable for studying the ultrathin-film heterostructures in that (massless) photons would deliver minimal impact to the specimens.^38,39^

In this work, we employed X-ray absorption spectroscopy (XAS) to examine the chemistry and the local electronic structure at the interface of the RS system. For the specimen, we chose a representative RS system composed of a Ta_2_O_5_ switching film and a Ta metal electrode.^15,18,40^ We compared this device with identical devices with inserted C layers of thicknesses ranging from a monolayer and 2 nm, sandwiched between them, i.e., Ta_2_O_5_/C/Ta. XAS has a probing depth larger than 5 nm, which would be long enough to catch the signals from the deep interfaces buried under the metal layers.^41^ Furthermore, XAS can reveal the bonding states of each atomic species so that the chemical environments at each layer can be identified unambiguously.^42,43^ The results of the XAS analyses combined with those of X-ray photoelectron spectroscopy (XPS) data show that the addition of C between Ta_2_O_5_ and Ta films induced a significant evolution in chemistry and electronic structure at the interface region, confirming the chemical functionality of the C interlayers in the representative RS system.

## Experimental Methods

### Sample Preparation

The Ta_2_O_5_/C/Ta samples were prepared by consecutive reactive sputtering steps of the respective layers without breaking the vacuum. Details of the growth process can be found elsewhere.^18,29^ First, 30-nm-thick Ta_2_O_5_ films were prepared by reactive alternating-current (AC) magnetron sputtering on a platinized SiO_2_/Si substrate. Then, C layers were deposited using ac magnetron sputtering. The thickness of the C layers was 2 nm for all samples (hereafter, written as “C”), except for a graphene-like C monolayer (hereafter, written as “Gr”). Ta is deposited onto the C layers by the same reactive sputtering (as that for Ta_2_O_5_) with targeted thicknesses of 5, 10, and 20 nm, while no external O_2_ was introduced during the Ta deposition. The actual thicknesses of the Ta films increased due to interfacial oxidation, which is closely related to the major finding in this work. For comparison, the Ta_2_O_5_/Ta films with the same Ta_2_O_5_ and Ta thicknesses were also prepared (without C) under the same conditions.

### XAS

XAS was conducted to scrutinize the unoccupied electronic structures of the Ta, O, and C atoms. Hard XAS at the Ta L_3_-edge (photon energy, *h*ν ∼ 9.9 keV) was conducted at the 8C beamline of Pohang Light Source (PLS) in a fluorescence yield mode. The incidence angle of the hard X-rays was set to 4° to maximize the signals from the Ta_2_O_5_ and Ta layers because the probing depth far exceeds the thicknesses of those layers.^44^ Meanwhile, soft XAS at C K-edge (*h*ν ∼ 290 eV) and O K-edge (*h*ν ∼ 530 eV) was done at the 2A beamline of PLS in a total electron yield mode. The incident X-rays were set perpendicular to the sample planes to maximize the probing depth (∼10 nm) to reach the interface region.^45^ In particular, O K-edge XAS is well-known to reflect the unoccupied electronic structure of oxides with a minimal core hole effect so that it is useful to investigate the conduction-band (CB) features. Combined with the valence-band (VB) XPS spectra, therefore, the O K-edge XAS spectra can provide a complete set of information on the electronic structure (both VB and CB) near the Fermi level.

XPS was performed with a Sigma Probe (ThermoVG) machine equipped with a monochromatic Al Kα source (photon energy, *h*ν = 1486.6 eV) in the National Center for Interuniversity Research Center at Seoul National University. The spectra reflect the electronic structure of a few top layers (within ∼2 nm) beneath a thin top surface of Ta (∼2 nm), which is almost fully oxidized due to air exposure. Synchrotron XPS at lower photon energies (*h*ν = 320 and 630 eV) was performed at the 8A2 (presently 10A2) beamline of PLS, equipped with a SES100 hemisphere analyzer (Omicron Inc.) to confirm the depth profile near the top layers (see the Supporting Information).

## Results and Discussion

### Cyclic Voltammetry (CV)

**Figure 1:**
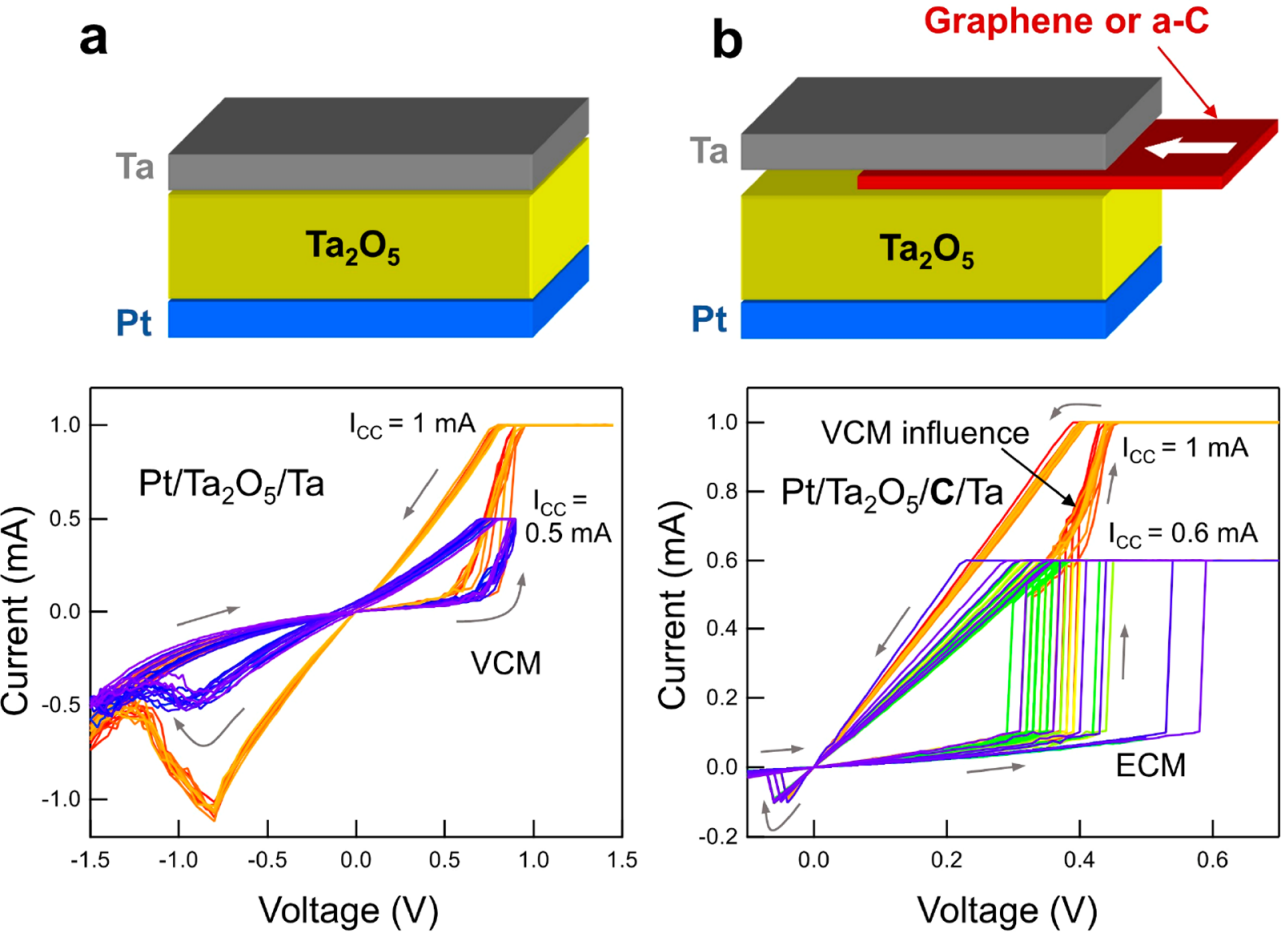
Schematics and CV curves for the (a) Ta_2_O_5_/Ta and (b) Ta_2_O_5_/C/Ta RS systems. The RS without C showed the typical VCM behavior, regardless of *I*_CC_, all of the time during the sweeps, while the RS with intervening C exhibited a combinational VCM + ECM behavior for a high *I*_CC_ (=1 mA) but had a pure ECM behavior for a low *I*_CC_ (=0.6 mA).

The RS behavior of C-intervened Ta_2_O_5_/Ta is fundamentally different from that without C. As discussed in previous studies, the addition of a graphene monolayer or a 2–3 nm thin amorphous C film causes a transition from VCM to ECM behavior, also observable as an abrupt change in the shape of the *I*–*V* sweeps.^9,40^ The upper panels of Figure 1a,b show the schematics of the tested sample structures with and without C layers, in which C is either graphene or 2-nm-thick amorphous carbon (a-C). The bottom panels show the CV curves of these devices, Ta_2_O_5_/Ta and Ta_2_O_5_/(2 nm a-C)/Ta, respectively. All of the sweeps in the Ta_2_O_5_/Ta system shown in Figure 1a indicate a typical VCM shape, regardless of the number of sweeps or the compliance current (*I*_CC_). However, adding 2 nm a-C as an intermediate layer results in current–voltage (*I*–*V*) characteristics, with an abrupt increase of the current at some threshold voltages during the SET reflecting the presence of an ECM in the C-added system. Meanwhile, as seen in Figure 1b, the linear decrease of the current in the ON state and sharp RESET are symptomatic for ECM/CBRAM.^9,40^

Interestingly, in C-added devices, sweeps at a higher compliance (*I*_CC_ = 1 mA) show an influence of VCM behavior during the SET event, whereas in the sweeps with a lower compliance (*I*_CC_ = 0.6 mA), this effect is absent. We explain the effect at higher compliance with the higher currents (and related Joule heating) in the device, allowing both cations and anions to pass through the C layer. Nevertheless, the RESET for both compliances is typical for ECM, indicating that a metallic (or metal-rich) filament is still dominating the switching mechanism. The essentially different electrical behavior from the reference Ta_2_O_5_/Ta samples was ascribed to the intervening C layers. Therefore, the chemistry of the interfacial C should be scrutinized by utilizing precision techniques in order to clarify the role of the C layers.

### Ta L-Edge XAS

**Figure 2:**
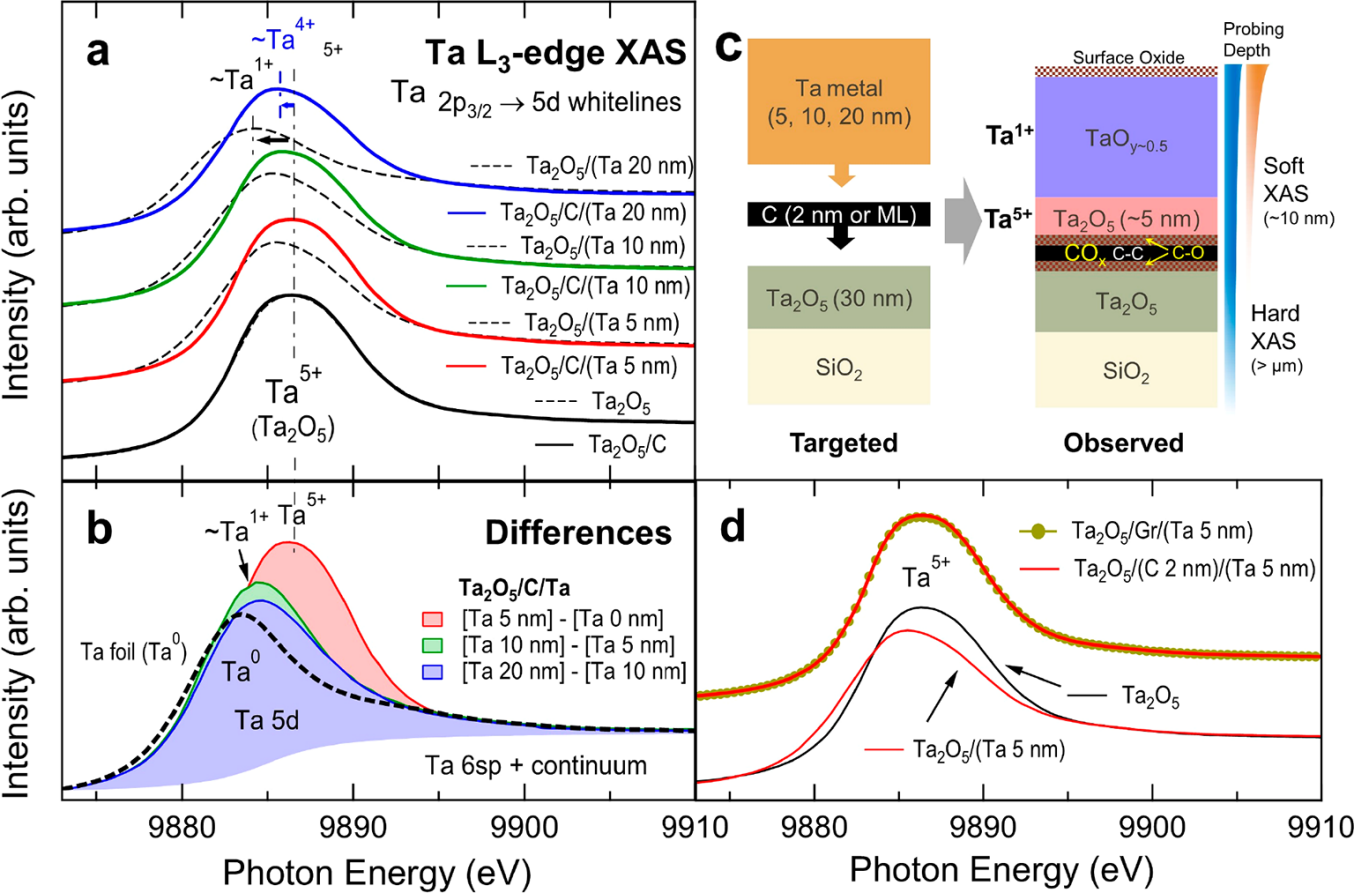
(a) Ta L_3_-edge XAS spectra of Ta_2_O_5_/Ta with or without C interlayers (2 nm), highlighting significant oxidation of the interfacial Ta layers in the C-added films. (b) Difference spectra for the depth-resolved analysis on the Ta oxidation states. (c) Schematics of the as-targeted and as-observed compositions. (d) Ta L_3_-edge XAS of the 2-nm-thick and monolayer (graphene) C samples.

Figure 2a shows the Ta L_3_-edge (2p_3/2_ → 5d or s-continuum) XAS spectra of the Ta_2_O_5_ (30 nm)/C (2 nm)/Ta (0, 5, 10, and 20 nm) films (solid curves). For comparison, appended are the spectra of the films with the same Ta_2_O_5_ and Ta thicknesses but without C layers (dotted curves). The peaks at *h*ν = 9883–9887 eV, called “white-lines” (WLs; 2p_3/2_ → 5d), represent mostly the unoccupied Ta 5d states, whereas the high-energy plateaus (>9895 eV) are mostly Ta 6sp and the higher quantum numbers’ continuum states. The energy and intensity of the WLs can be utilized to identify the average chemical states of Ta_2_O_5_ and Ta.^46,47^ With increasing oxidation number, the WL energy increases from ∼9883 eV (Ta^0^) to ∼9887 eV (Ta^5+^), and the areal intensity of the WLs gradually increases according to the emptiness of the 5d orbital states.^18,48^

A linear interpolation of the WL energies to the Ta valence from Ta^0^ (9883 eV) to Ta^5+^ (9887 eV) tells that, in the case of Ta_2_O_5_/Ta, the average Ta valence changes gradually from 5+ to ∼1+ with increasing Ta thickness, obviously showing the effect of Ta addition. Note that, even for the 20-nm-thick Ta sample, the Ta valence is not zero but ∼1+, suggesting partial oxidation, TaO_*y*∼0.5_, in the Ta layers. On the other hand, in the case of Ta_2_O_5_/C/Ta, the Ta valence still decreases but with a considerably smaller amount (from 5+ to 4+ only). The measured average valence for the 20 nm Ta sample is 4+, much larger than 1.9+, which is the value from the nominal average of 30 nm Ta_2_O_5_ and 20 nm Ta.^49^ Also, the WLs are overall more intense than those for Ta_2_O_5_/Ta, suggesting overall higher densities of the unoccupied states, i.e., higher valences.^48^ These evidently show that the Ta layers in the C-added samples are oxidized significantly.

It should be noted that the spectrum of Ta_2_O_5_/C almost coincides with that of Ta_2_O_5_. This indicates that the chemistry of the bottom Ta_2_O_5_ layer does not change due to the C deposition process. Then the significant difference in Ta chemistry between Ta_2_O_5_/C/Ta and Ta_2_O_5_/Ta should be understood as such in that the presence of the C interlayers expedites oxidation of the Ta metal layers.

In order to scrutinize the role of C layers in Ta oxidation, the depth profile of the oxidation state of the Ta layers was examined using a difference analysis,^18,50,51^ in which the spectral evolution with increasing Ta thickness was interpreted as the contribution of the added Ta layers. For instance, the signals from the first 5 nm Ta on the C layers can be presumed as the difference between the spectra of Ta_2_O_5_/C/(Ta 5 nm) and Ta_2_O_5_/C, i.e., [Ta 5 nm] – [Ta 0 nm], and the signals from the next 5 nm Ta can be done as [Ta 10 nm] – [Ta 5 nm], etc.

The difference spectra are displayed in Figure 1b. These reflect the depth profiles of the oxidation states in the added Ta. That is, [Ta 5 nm] – [Ta 0 nm] reflects the chemistry of the first 5 nm Ta, whereas [Ta 10 nm] – [Ta 5 nm] and [Ta 20 nm] – [Ta 10 nm] are the chemistry of the next 5 nm Ta and last 10 nm Ta, respectively. It can be observed that the WL energy of [Ta 5 nm] – [Ta 0 nm] remained at ∼9887 eV, the energy for Ta^5+^. This indicates that the first 5 nm Ta is fully oxidized (Ta_2_O_5_). Meanwhile, the WL energies of the [Ta 10 nm] – [Ta 5 nm] and [Ta 20 nm] – [Ta 10 nm] spectra become ∼9884 eV, the energy for Ta^+^, rather than ∼9883 eV for Ta metal (see the spectrum of a Ta foil appended in the figure). This indicates that the Ta layers (except for the first 5 nm Ta) are partially oxidized as in TaO_∼0.5_.

**Figure 3:**
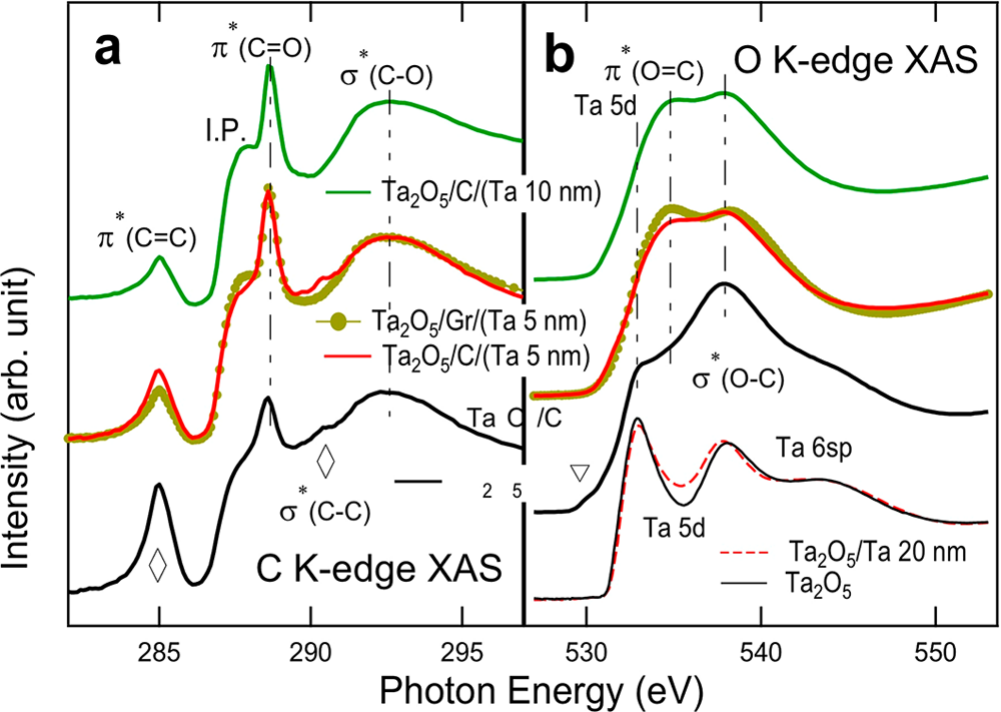
(a) C K-edge and (b) O K-edge XAS spectra of the Ta_2_O_5_/C/Ta system showing the prevalence of C–O bonds.

Figure 2c depicts the findings in the Ta L_3_-edge XAS, full oxidation of the first 5 nm Ta, and partial oxidation of the next 15 nm Ta, together with the observations from C K-edge and O K-edge XAS (Figure 3). The observed depth profile of the Ta_2_O_5_/C/Ta heterostructure appears to be quite different from the targeted compositions. The higher degree of Ta oxidation (Ta^5+^) in the Ta layers near the C interlayers, in contrast to Ta^+^ in the Ta layers above, implies that the oxidation must be closely related to the C layers.

Apart from the Ta oxidation profile, two additional points are to be addressed. One is that the C interlayers themselves are also oxidized to form C–O bonds. The presence of C–O bonds will be evidenced in C K-edge and O K-edge XAS (Figure 3). This clearly shows that oxidation of the Ta metal layers is indeed incorporated by chemical bonding with O atoms. That is, the C layers become CO_*x*_, in fact, which then can serve as a donor of oxygen toward the Ta layers above.

The other point is that, for the Ta-added samples, the top surface (within ∼2 nm depth from the surface) is also oxidized due to air exposure. In the case of XPS, the signals from the surface oxide are very intense and have constant binding energies (BEs), so that they could be used for the BE calibrations (Figure S1). Also, surface oxidation occurred for all of the samples, so that its effect was canceled out in the difference spectra shown in Figure 2b. Meanwhile, in the case of XAS, the signals from the very surface are hardly noticeable because of its much longer probing depth (at least 10 nm).

Figure 2d highlights the difference in the Ta L_3_-edge XAS spectrum between the Gr- and C-added samples (with 5 nm Ta on top). The spectra of a bare Ta_2_O_5_ film (Ta^5+^) and the Ta_2_O_5_/Ta 5 nm film (Ta^∼4+^) are appended for comparison. The spectra almost coincide with each other and the spectrum of a bare Ta_2_O_5_ film, suggesting full oxidation (Ta^5+^) of the first 5 nm Ta for both Gr- and C-added films. Thus, the graphene-like interlayer appears to play almost the same function as that of the 2 nm C layer despite its smaller volume. Therefore, it can be concluded that the C addition expedites oxidation of the Ta metal, particularly the Ta layers nearby (within ∼5 nm distance).

### C K-Edge and O K-Edge XAS

The information on the chemical bonding states of the C and O atoms is revealed in Figure 3, which shows the soft XAS spectra of the C- or Gr-added films taken at the (a) C K-edge and (b) O K-edge, which reflect the local coordination of the C and O atoms, respectively. Unlike the case of hard XAS (Figure 2), soft XAS has a relatively short probing depth (∼10 nm; Figure 2c)^45^ so that the captured signals from the C or Ta metal layers are much stronger than the signals from Ta_2_O_5_ beneath the C layers.

Each of the features in the C K-edge XAS spectra can be assigned according to the C-bonding-related excited states.^43,52−54^ In the order of increasing *h*ν values, the peaks near *h*ν = 285, 289, 290, and 292 eV in Figure 3a can be attributed to the excited states of π*(C=C), π*(C=O), σ*(C–C), and σ*(C–O), respectively,^55−57^ whereas the intense shoulder at 287 eV can be attributed to the ionization potential (IP; transition to the vacuum level).^43,55^ The features related to the bonds among C atoms (C=C or C–C) are indicated by diamonds in the figure.

Even for Ta_2_O_5_/C (without Ta), the features of the π*(C=O) and σ*(C–O) excited states are clearly observed, indicating that the C–O bonds prevail (in its ground state) in the C layers. This suggests that the C layers are readily oxidized to form CO_*x*_ in part. It is shown in the O 1s XPS spectra (Figure S1) that the shoulder peak (near 533 eV, which accounts for the O–C bonds) of the Ta_2_O_5_/C sample is not very enhanced compared to that of the Ta_2_O_5_/C/Ta samples. This is evidence that surface oxidation of the C layers due to air exposure is much less significant than the case of Ta metal (Ta–O bonds). Therefore, the O^2−^ ions in the C–O bonds should be donated by Ta_2_O_5_ at the bottom, and it is justified to say that the C–O bonds are rich in the C layers close to the bottom oxide.

It is shown that, as the Ta thickness increases, the C–O-bond-related peaks become more prominent over the C–C-bond-related peaks. The thickness-dependent evolution in the peak intensities can be interpreted as the signature of different spatial distributions of C–O and C–C; that is, the C–O bonds are more abundant at the interface toward the Ta layers than inside the C layers. As the Ta thickness increases, C K-edge XAS with finite probing depth will capture the signals more from the shallower end (i.e., C–O regions), as depicted in Figure 2c. This is reasonable in that the Ta layers near the C layers are fully oxidized so that the C atoms near the oxidized Ta (or the bottom Ta_2_O_5_) would be more oxidized than the C atoms in the middle of the C layers.

It is noteworthy that the spectrum of Ta_2_O_5_/Gr/Ta 5 nm shows weak C–C bond features compared to the case of the Ta_2_O_5_/C/Ta 5 nm sample. This implies that the graphene-like monolayer was subjected to severe oxidation (due to the surrounding Ta oxides), leaving a smaller number of C–C bonds compared to 2 nm C in the other samples.

The O K-edge XAS spectra in Figure 3b reflect mainly the hybridized orbital states of O 2p + Ta 5d/6sp (from Ta oxide) or O 2p + C 2p (from CO_*x*_). The spectra of Ta_2_O_5_/(Ta 20 nm) and Ta_2_O_5_ (without C) are appended for comparison. From those spectra, the signatures of the O–Ta-bond-related hybridized orbital states can be identified as follows: O 2p + Ta 5d hybridized states (doublets at *h*ν ∼ 532 and 538 eV) and O 2p + Ta 6sp states (bumps at *h*ν ∼ 543 eV).^18^

It can be noticed at a first glance that the spectra of the C-added films are much different from those of the reference films (without C). In the case of Ta_2_O_5_/C or Ta_2_O_5_/C/Ta, additional features (other than those from O–Ta) can be attributed to the π*(O=C) and σ*(O–C) excited states at ∼534 and ∼538 eV, respectively.^43,55^ These assignments manifest that O is indeed incorporated in the oxidation of Ta and C. Furthermore, the addition of C interlayers significantly alters the chemical and electronic structure as well. The spectrum of Ta_2_O_5_/C shows an additional low-energy bump (empty triangle in Figure 3b), showing a metallic in-gap state. The metallicity might originate from the lightly oxidized graphite or graphene layers.^58,59^

### XPS

**Figure 4:**
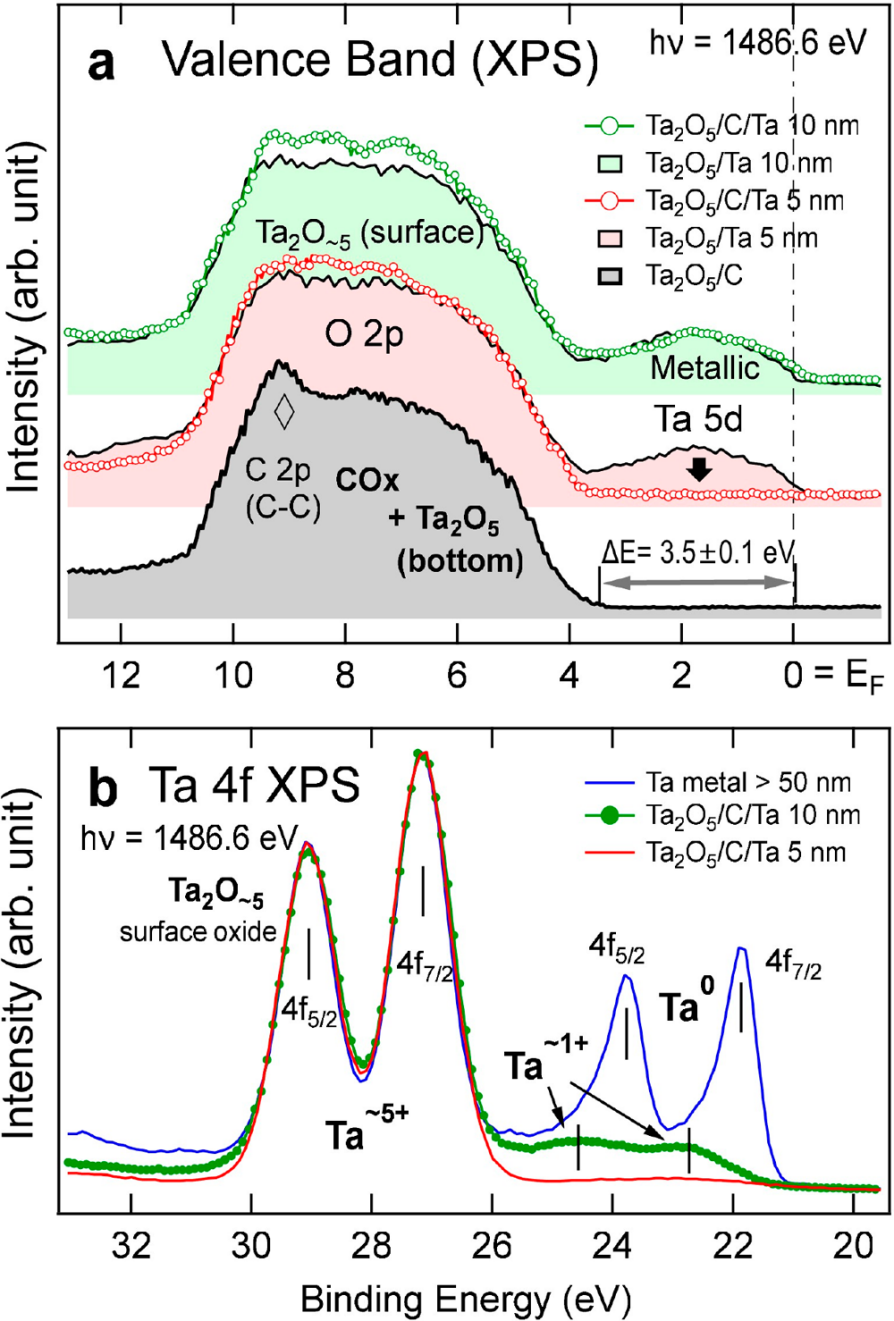
(a) XPS VBs and (b) Ta 4f core-level spectra. The spectra are contributed by surface oxides (Ta_2_O_∼5_), fully oxidized first 5 nm Ta (Ta_2_O_5_), and partially oxidized (TaO_*y*<1_) in the next Ta layers.

The electronic structure of the Ta_2_O_5_/C/Ta system is further scrutinized by XPS. Figure 4a shows the VB spectra of the Ta_2_O_5_/C, Ta_2_O_5_/C/Ta, and Ta_2_O_5_/Ta samples taken with a monochromatic Al Kα source (*h*ν = 1486.6 eV). The VB features consist of O 2p mainly from the Ta_2_O_∼5_ oxidized surface^60^ and CO_*x*_ (BE = 10–4 eV),^61^ C 2p from C–C bonds,^62^ and Ta 5d near the Fermi level (*E*_F_; BE = 0 eV) mainly from metallic Ta layers.^63^ For Ta_2_O_5_/C, the VB maximum (VBM), estimated by extrapolation of the VB to the abscissa, was at BE ∼ 3.5 eV, reflecting an insulating nature of the oxidized C layers (CO_*x*_). For the Ta_2_O_5_/Ta and Ta_2_O_5_/C/(Ta 10 nm) samples, on the other hand, the metallic Ta 5d states are shown beneath *E*_F_, indicating metallicity of the Ta layers (TaO_*y*∼0.5_).

Interestingly, the spectrum of Ta_2_O_5_/C/(Ta 5 nm) shows no Ta 5d feature near *E*_F_, suggesting an insulating nature of the fully oxidized first 5 nm layers (Ta^5+^; d^0^), which is consistent with the results of Ta L_3_-edge XAS in Figure 2. This is in contrast to the persistent Ta 5d state in the spectrum of Ta_2_O_5_/(Ta 5 nm), evidencing that the added C greatly facilitates oxidation to open the band gap. The VBM of Ta_2_O_5_/C/(Ta 5 nm) was estimated to be at BE ∼ 4.0 eV.

The Ta 4f XPS spectra of Ta_2_O_5_/C/Ta and a thick Ta metal reference (>50 nm) are shown in Figure 3b. The 4f_5/2_–4f_7/2_ doublet feature at BE = 29.1 and 27.1 eV can be attributed to Ta^5+^ mostly from the surface oxide, whereas the small lower-BE shoulder peaks can be attributed to less oxidized Ta ions such as Ta^+^ or Ta^0^ (BE= 25–21 eV) in the Ta metal layer.^64^ It is shown that, even for a thick Ta metal film, the surface oxidation is so severe that the signature of Ta^5+^ from the surface oxidation appears to be bigger than that of Ta^0^, making the chemical assessment via XPS challenging. For the Ta_2_O_5_/C/(Ta 10 nm) sample, 4f_5/2_–4f_7/2_ shoulder peaks appear near BE = 24.8 and 22.8 eV, indicating the presence of Ta^+^ at the top Ta layers (beneath the surface oxide). For the Ta_2_O_5_/C/(Ta 5 nm) sample, the shoulder peaks almost disappeared, confirming full oxidation of the first 5 nm Ta. Synchrotron XPS using lower *h*ν values further showed that the shoulder peaks (Ta^+^) get weaker as *h*ν decreases (Figure S2), confirming that TaO_*y*<1_ are indeed buried under the surface oxides.

### Electronic Structure

**Figure 5:**
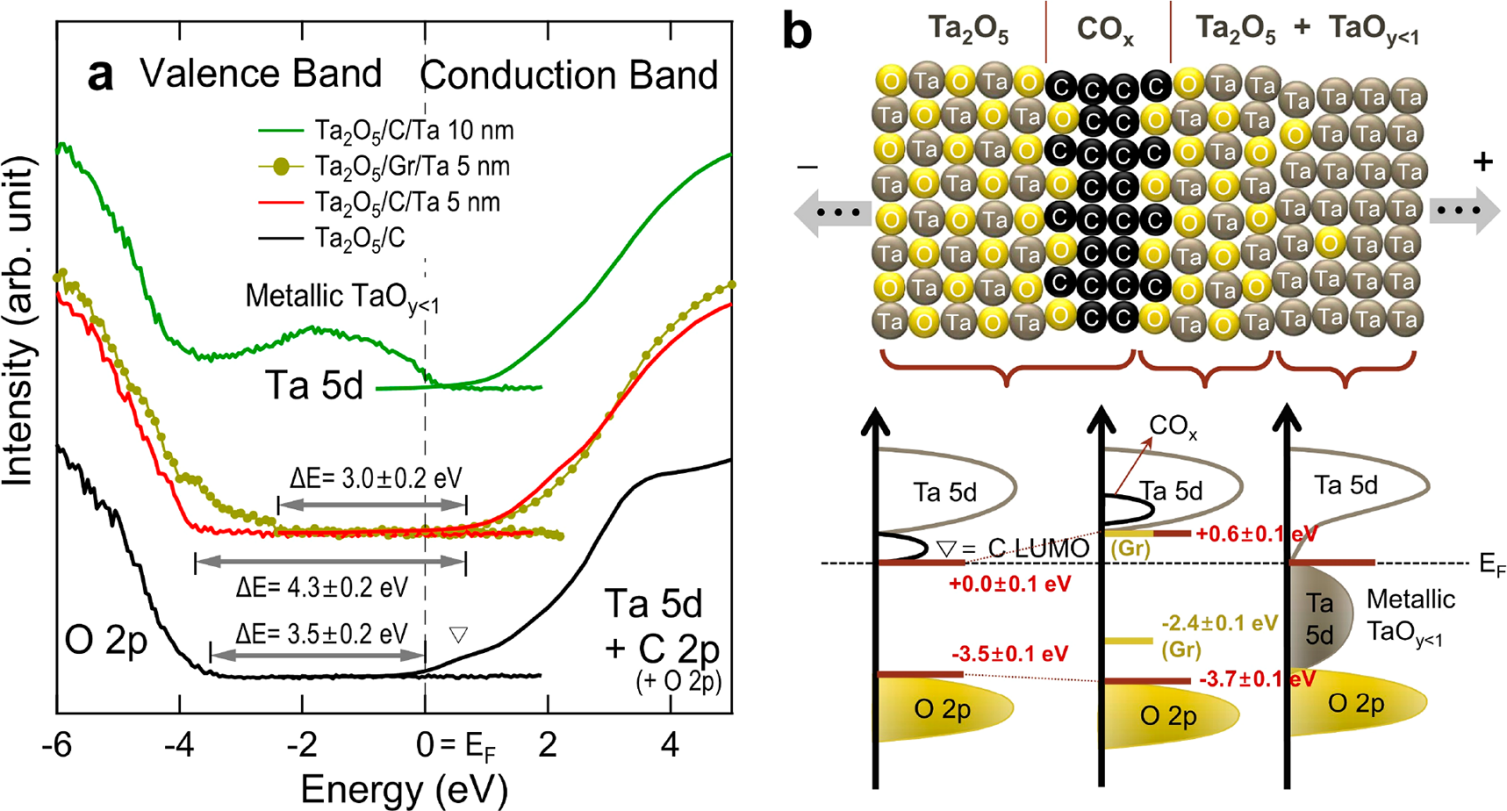
(a) Combined VB (XPS) and CB (O K-edge XAS) spectra of the Ta_2_O_5_/C/Ta system. (b) Schematic sample structure (upper panel) and the constructed energy diagram (lower panel) for the depth-resolved electronic structure. The CBM and VBM are indicated by the bars in the lower panel. Overall error values account for the uncertainties in determining the energies of the CBM/VBM.

Figure 5a shows the combined XPS VB and O K-edge XAS spectra near the band gap as functions of energy relative to *E*_F_. Generally, the O K-edge XAS line shape can be safely assumed to represent the CB structure because the interaction between the O 1s core hole and the cation’s orbital state is minimal.^65^ However, the values of *h*ν in the XAS data should be converted into energy relative to *E*_F_. It is shown in the O 1s XPS data (Figure S1) that the main peaks (O^2–^ in Ta_2_O_5_, mainly the surface oxide) for all of the Ta_2_O_5_/C/Ta samples remain at a fixed BE (∼531 eV). The identical BE for the O 1s core levels suggests that possible sample dependence in the O^2–^ chemistry or in the *E*_F_ values is negligible.^66^ Therefore, all of the O K-edge XAS spectra were shifted rigidly by ∼−530 eV to fit the energy difference between the CB minimum (CBM) and VBM of Ta_2_O_5_ to the experimental value of the band gap (∼4.0 eV).^67,68^

In the case of Ta_2_O_5_/C, the CB protrudes toward and touches the *E*_F_ (highlighted by the empty triangle). Because the CB tail should originate from the lowest unoccupied molecular orbital (LUMO) states of the C layers,^69^ it reflects the metallicity of the partially oxidized C layer (CO_*x*_; Figure 2c). The band gap (Δ*E*), namely, the energy difference between the CBM (pinned at 0 eV) and VBM, is estimated to be approximately 3.5 eV.

In the case of Ta_2_O_5_/(a-C 2 nm)/(Ta 5 nm), the top Ta layers are fully oxidized and have Δ*E* ∼ 4.3 eV. At the same time, the tail state due to CO_*x*_ becomes invisible probably because of further oxidation, as is evidenced in Figure 3. In the case of Ta_2_O_5_/Gr/(Ta 5 nm), the band gap becomes 3.0 eV, which is much smaller than 4.3 eV (a-C), and the VBM is lifted over +1 eV. The small band gap in the Gr sample compared to the a-C sample might be relevant to the high conductance of graphene oxide.^59^ Meanwhile, in the case of Ta_2_O_5_/(C 2 nm)/(Ta 10 nm), the top Ta layers are oxidized only in part, so that the metallic Ta 5d state remains and touches *E*_F_.

All of the electronic structural information obtained by the XPS and O K-edge XAS data is summarized in Figure 5b. In the upper panel, the depth-resolved schematic for the composition according to the results in Figures 2–4 is displayed. The C interlayers fully oxidize the adjacent Ta layers (∼5 nm) to constitute a physical barrier.^29^ At the same time, the C layers themselves are oxidized as well to form C–O bonds (CO_*x*_). The depth-resolved electronic structures of CB (Ta 5d or C LUMO) and VB (O 2p or Ta 5d), together with the CBM/VBM (bars), are also depicted in the bottom panel.

The influence of C addition in the Ta_2_O_5_/Ta RS on the interfacial electronic structure can be concluded as follows. The C layers are not inert but chemically active so as to not only form C–O bonds but also significantly oxidize the adjacent Ta layers. The fully oxidized Ta layers (within ∼5 nm distance) are found to be insulating with a band gap of >4 eV to serve as a physical barrier. Meanwhile, the Ta layers far from the C layers are partially oxidized (TaO_*y*<1_) and still serve as a metallic component in the RS devices.

According to the reports on the VCM mechanism in Ta-oxide-based RS,^18,23^ a partially reduced matrix environment as in TaO_2_ appears to be essential for promoting the VCM activities for the coalescence of oxygen vacancies and would lead to formation of the conducting filaments. Then, transformation of the first 5 nm Ta near the C layers into a stoichiometric Ta_2_O_5_ will mitigate the VCM/OxRAM activities across the Ta_2_O_5_/C/Ta_2_O_5_/TaO_*y*_ RS cells. Meanwhile, the ECM/CBRAM activities of the cations (Ta ions) would not be suppressed by the oxidized interface. Therefore, the C-added RS cells would suffer an apparent VCM-to-ECM transformation subject to the activity of C interlayers, consistent with the CV results (Figure 1).

This work demonstrates that the combined X-ray spectroscopies (XPS and XAS) study can reveal the role of an artificially added interlayer on the readily active oxide matrix/metal electrode resistive switch in terms of the structural, chemical, and electronic structural evolution of each layer. The layer-dependent electronic structure of the C(Ox) interlayer or the Ta metal layers (TaO_∼2.5_ near the interface and TaO_*y*<1_ far from the interface) clarifies that the changes in the structural and chemical environment by C insertion at the interface can indeed induce abrupt changes in the electronic structure near both the VBM and CBM (Figure 5).

The variety in the energy levels summarized in Figure 5b implies that the RS characteristics can also vary delicately depending on the details in the C-added structures. In this regard, insertion of the chemically active C interlayers would provide a novel tuning knob for controlling the electrochemical properties not only in RS devices but also in general oxide-metal-interface-based electronic devices via interfacial oxidation, evoking the strong need for further studies on the chemical activity of the graphite/graphene interlayer.

## Conclusions

The combined XAS and XPS study on the graphene (or amorphous C)-layer-added Ta_2_O_5_/Ta resistive switches shows that the presence of C significantly facilitates oxidation of the adjacent Ta metal electrode. The first 5 nm Ta from the interface toward C becomes fully oxidized, transforming to a large band-gap (∼4.3 eV) insulator, while the next 15 nm Ta far from the C interface remains metallic, being partially oxidized (TaO_*y*<1_). The depth-resolved quantitative information on the electronic structure, which is analyzed in terms of the respective orbital states (Ta 5d, O 2p, and C LUMO), clearly demonstrates the functionality of the added C layer as an oxidizing agent. The fully oxidized Ta metal layers act as physical barriers to suppress the VCM mechanism, leaving ECM dominant for RS. The insertion of C layers would benefit engineering of the RS characteristics at the oxide/metal interface, while exposure to air in growth processes of the RS system might be detrimental for controllability. This work highlights the importance of identifying the chemical interactions and effects of the intermediate layers in determining the mass and charge transport through interfaces and the related changes in the device functionalities.

## Supporting Information Available

Ta 4f and O 1s XPS spectra of Ta_2_O_5_/C/Ta films (PDF)

## Abbreviations

ECM: electrochemical metallization memory
RS: resistive switching
VCM: valence change memory
XAS: X-ray absorption spectroscopy
XPS: X-ray photoelectron spectroscopy
