# Supplementary material for: Chemical Influence of Carbon Interface Layers in Metal/Oxide Resistive Switches
Source: ACS Appl Mater Interfaces. 2023 Mar 29;15(14):18528–36. doi: 10.1021/acsami.3c00920 (PMC10103050; doi:10.1021/acsami.3c00920)
Supplement: Supplementary file 1 — am3c00920_si_001.pdf [file am3c00920_si_001.pdf]

Supporting Information

Chemical Influence of Carbon Interface Layers in Metal-Oxide Resistive Switches

*Deok-Yong Cho†, Ki-jeong Kim ||, Kug-Seung Lee ||, Michael Lübben#, Shaochuan Chen‡, Ilia Valov#,§,**

†IPIT and Department of Physics, Jeonbuk National University, Jeonju 54896, Korea, || Pohang Accelerator Laboratory, Pohang 37673, South Korea, #Peter Gruenberg Institute, Research Centre Juelich, Juelich 52425, Germany, ‡IWE2, RWTH Aachen University, Sommerfed str.24, Aachen 52074, Germany, §Institute of electrochemistry and energy systems "acad. E. Budewski", Bulgarian academy of sciences, "acad. G Bonchev" str. Bl.10, 1113 Sofia Bulgaria

*e-Mail: i.valov@fz-juelich.de

1. O 1s XPS spectra

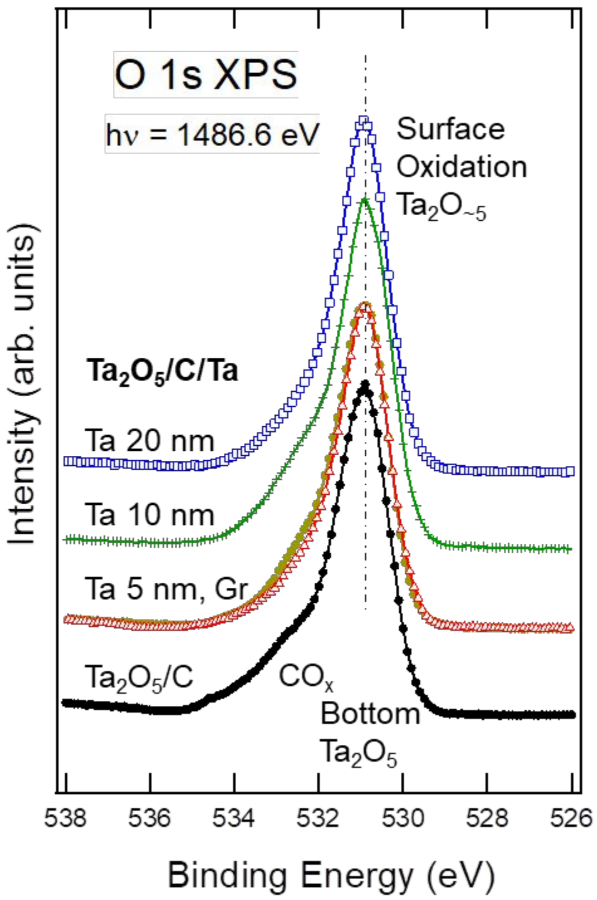

Fig. S1. The O 1s XPS spectra (hν= 1486.6 eV) of the Ta_2O_5/C and Ta_2O_5/C/Ta samples. The most intense O 1s peaks (~531.0 eV) originate from the surface oxide (due to surface oxidation of Ta). The identical binding energy (BE) for all the main peaks suggests that chemical distinctions or Fermi level shifts among the (oxidized) sample surfaces can be hardly noticed. Therefore, the conduction band features can be deduced from the O K-edge XAS data (Fig. 2a)

after an arbitrary rigid shift (~-530 eV, to compensate the O 1s core level energy) as shown in Fig. 4a in the text.

2. **Photon-energy-dependent Ta 4f XPS spectra**

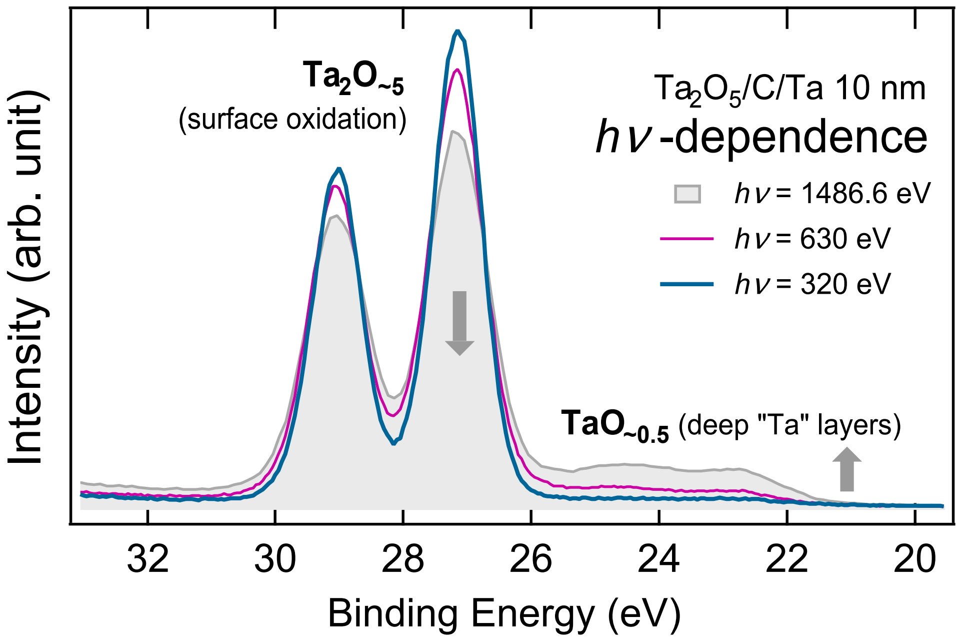

Fig. S2. The Ta 4f XPS spectra of Ta_2O_5/C/Ta 10 nm sample taken at three different photon energies (hν= 320 eV, hν= 630 eV and hν= 1486.6 eV). The main 4f doublets at BE= 26 to 30eV are from the surface Ta-oxide (from surface oxidation), whereas the 4f doublets at BE= 22 to 26 eV are from the top Ta metal layers beneath the surface, which are slightly oxidized ($TaO_{y<1}$). As

the hν increases, the signature of TaO_y becomes more prominent in contrast to that of the surface oxide. The hν-dependence originates from the difference in probing depth: For hν= 320 eV data, the signals are from the oxidized Ta at the top layers (~ 2 nm) mostly, while for hν= 1486.6 eV data, the signals from the deeper regions, i.e. the Ta metal layers ($TaO_{y\sim0.5}$), are also captured. Hence, it is confirmed that the Ta^{1+} signals from the Ta metal layers are persistent despite the air contamination at the top surface.
